# Influence of Bentonite on Mechanical and Durability Properties of High-Calcium Fly Ash Geopolymer Concrete with Natural and Recycled Aggregates

**DOI:** 10.3390/ma14247790

**Published:** 2021-12-16

**Authors:** Rana Muhammad Waqas, Faheem Butt, Aamar Danish, Muwaffaq Alqurashi, Mohammad Ali Mosaberpanah, Bilal Masood, Enas E. Hussein

**Affiliations:** 1 Civil Engineering Department, University of Engineering & Technology, Taxila 47080, Pakistan; 2 Civil Engineering Department, Faculty of Engineering Sciences, National University of Modern Languages, Rawalpindi 04631, Pakistan; aamar.danish@numl.edu.pk; 3 Department of Civil Engineering, College of Engineering, Taif University, Taif 21944, Saudi Arabia; m.gourashi@tu.edu.sa; 4 Civil Engineering Department, Cyprus International University, Nicosia 99258, Turkey; mmosaberpanah@ciu.edu.tr; 5 Civil Engineering Department, Wah Engineering College, University of Wah, Wah Cantt 47040, Pakistan; bilal.masood@wecuw.edu.pk; 6 National Water Research Center, P.O. Box 74, Shubra El-Kheima 13411, Egypt

**Keywords:** geopolymer concrete, bentonite, recycled aggregates, GRAC, GNAC, mechanical properties, durability, rheological properties

## Abstract

In this study, bentonite (a naturally occurring pozzolana) was incorporated as a partial replacement (up to 20%) for high-calcium fly ash (HCFA)-based geopolymeric natural aggregate concrete (GNAC) and geopolymeric recycled aggregate concrete (GRAC). The mechanical (compressive strength and splitting tensile strength), durability (chloride migration coefficient, water absorption, and acid attack resistance), and rheological properties (slump test, fresh density, and workability) were investigated. The results revealed that incorporation of bentonite (10 wt % with ordinary Portland cement) showed appreciable improvement in the strength and durability of both the GNAC and GRAC, though its effect is more significant for GRAC than the GNAC.

## 1. Introduction

Concrete is an abundantly used material in the construction industry due to its distinctive properties such as cost-effectiveness, high strength, durability, easy availability of its constituent materials, and ease of formability into desired shapes. Ordinary Portland cement (OPC) is the main constituent of concrete necessary to bind aggregates together [1]. However, the production of OPC requires an appreciable amount of energy and generates approximately 10% of total carbon dioxide (CO_2_) emissions worldwide, which is one of the main reasons for global warming [2]. According to an estimate, about 2.8 billion tons of cement are produced each year and will reach 4 billion tons by 2050 [3]. Over the past two decades, constructional activities have grown rapidly around the globe due to increased infrastructure demand arising from the increase in global population and urbanization. Accordingly, the production of cement is also increasing to meet the increased demand for infrastructure. The production of cement causes serious damage to the environment due to greenhouse gas (GHG) emissions. Therefore, serious environmental concerns have been raised regarding the production of OPC [4]. To address such concerns, researchers are suggesting partial or full replacement of OPC in concrete construction with supplementary cementitious materials (SCMs) [2]. Due to urbanization and infrastructure developments, there is a significant increase in the generation of construction and demolition waste (C&DW) materials resulting from the demolition of old infrastructures [5]. Moreover, the extraction of natural coarse aggregates (NCA) involves a large amount of energy and depletion of natural resources, which is affecting the eco-system. Therefore, to dispose of and manage these wastes (C&DW) and develop alternatives to traditional OPC has become an essential requirement for sustainability. Consequently, researchers are trying to find new ways of recycling C&DW to save natural aggregates reserves and reduce environmental pollution [6,7,8]. The fine and coarse aggregates constitute 75–80% of concrete’s total volume and C&DW can be used as recycled coarse aggregates (RCA) in new concrete to minimize the consumption of natural resources that are used to produce NCA [9]. However, the studies have found that concrete incorporating RCA produces inferior mechanical and durability properties than concrete containing NCA [10,11,12,13,14,15,16,17]. It was reported by Etxeberria et al. [8], Sagoe Crenstil et al. [9], and Tabsh and Abdelfatah [11] that OPC concrete containing 100% RCA has 10–40% lower compressive strength than the concrete containing NCA. Moreover, Marinkovic et al. [12] reported that the incorporation of RCA in OPC concrete leads to an increase in the absorption capacity and drying shrinkage than the concrete with NCA. This is due to the porous nature, low density, and high-water absorption of old cement mortar attached to the surface of RCA [13,14,15]. Similarly, the use of electric arc furnace (EAF) slag and ground granulated blast furnace slag (GGBFS) as partial or total substitution of natural aggregates (NAs) in structural concrete elements have also been studied by different researchers. This material is a byproduct of steel production in electric arc furnace plants [18,19]. Domenico et al. studied the use of EAF as partial replacement of coarse aggregates in reinforced concrete (RC) beams [18]. They found that the presence of EAF led to a higher flexural and shear capacity than the corresponding traditional RC beams, crack widths are reduced, and the overall ductility is increased. The use of waste materials such as C&DW, EAF, and GGBFS as aggregates in concrete will lead to a sustainable and economical practice in the construction industry.

Geopolymer is an emerging and innovative binder that can provide an environmentally friendly replacement of OPC. The materials with geological origin, i.e., metakaolin and by-products such as fly ash (FA), ground granulated blast furnace slag (GGBS), and rice husk ash (RHA), chemically activated by alkaline solution (sodium silicate and sodium hydroxide) are generally used to synthesize geopolymer. It has been estimated that geopolymer binder generates 80% less CO_2_ emissions and requires 60% less energy than the manufacturing OPC [20]. Extensive research has been carried out on the development of geopolymer as a sustainable binder [21,22,23,24]. The incorporation of RCA in the geopolymer concrete (GPC) is a step to further improve environmental sustainability. Some recent studies have revealed that the negative effects of RCA on mechanical and durability properties of GPC are similar to that of the conventional concrete with RCA [25,26].

To improve properties of the geopolymeric recycled aggregate concrete (GRAC) various methods are suggested, e.g., increasing the fineness of fly ash in geopolymers to improve the degree of geopolymerization [27,28], and incorporation of calcium-rich materials such as OPC and GGBS to enhance strength (early age) and setting time [29,30]. Some studies have suggested incorporating SiO_2_-rich materials (rice husk ash, nano SiO_2_) that improve the strength and durability of the resulting GRAC [31,32]. Nuaklong et al. [33] reported the use of metakaolin, a mineral admixture, to improve the mechanical and durability properties of high-calcium fly ash-based geopolymer concrete containing RCA.

The low-calcium bentonite is a naturally found pozzolana, rich in silica and alumina, that has been extensively used for many years to improve the mechanical and durability properties of conventional concrete [34,35]. Bentonite is an alumino-phyllosilicate clay, comprising mainly montmorillonite minerals and usually formed by the chemical weathering of volcanic ash in the presence of water. The use of bentonite clay as a partial replacement of cement in conventional concrete containing both natural and recycled coarse aggregates has been widely reported [36,37,38,39,40,41,42,43]. The industrial bentonites are generally either the sodium or calcium variants. Bentonites have excellent rheological and absorbent properties. Among various types of bentonites, calcium bentonite has a much lower swelling capacity [36]. Bentonite is found in many regions of Pakistan, including Attock, Jhelum, Nowshera, and Karak. Memon et al. [42] and Mirza et al. [43] have reported the use of low-calcium bentonite as a partial replacement of OPC. In previous studies, bentonite was used for mass replacement of OPC up to 21%. Bentonite mixes outperformed conventional/OPC concrete at 56- and 90-day strength, and resistance improvement against acid attack was also observed. Masood et al. [41] reported the use of low-calcium bentonite as a partial replacement of cement in OPC concrete containing RCA [43] and found that the incorporation of bentonite in concrete with RCA resulted in improved mechanical and durability properties compared to the concrete with NCA. The improved mechanical and durability properties of concrete are attributed to the high pozzolanic reactivity and filling effects of bentonite. In terms of geopolymer concrete (GPC), Ekaputri and Antoni [44] evaluated the effect of bentonite on the GPC containing geothermal silica. They cured the specimens for a specific amount of time at a temperature between 80–120 °C and then kept them at room temperature for 7 days. The results revealed that the addition of bentonite and higher initial curing temperature improved the mechanical properties of GPC. In another study by Yang et al. [45], porosity of geopolymer prepared with pre-swelled bentonite slurry was analyzed. They concluded that incorporation of bentonite reduces pore size in the matrix, leading to improved compressive strength and thermal insulation performance. Similarly, Huang [46] reported the influence of calcium bentonite on the compressive strength, aesthetics, and drying shrinkage of fly ash-based geopolymer mortar. They observed that incorporation of calcium bentonite improves the compressive strength of specimen owing to the increased ratio of silica/alumina (Si/Al). They also concluded that the addition of calcium bentonite reduced drying shrinkage of the specimen, attributed to the peculiar properties of calcium bentonite, i.e., volume expansion and water adsorption. Moreover, the study reported the elimination of efflorescence in calcium bentonite-modified geopolymer mortar due to the adsorption of sodium ion by calcium bentonite via ion exchange.

GPC has emerged as an innovative construction material with environmental benefits and better engineering properties. The addition of RCA in geopolymer concrete can be associated with a further increase in environmental sustainability. The geopolymeric recycled aggregate concrete (GRAC) has benefits of reducing the CO_2_ emissions owing to the production of cement and preservation of natural aggregate resources. Furthermore, GRAC can resolve issues associated with the disposal of large volumes of construction and demolishing wastes (C&DW). Despite several environmental benefits of GRAC, it has inferior engineering properties due to weak internal structure as compared to GNAC. The specific objective of the present study is to improve the mechanical and durability properties of GRAC mixes by incorporating SCM. The review of the existing literature indicates that low-calcium bentonite has promising effects on the mechanical and durability properties of conventional concrete [37,42,43,47,48,49,50]. However, the studies on the inclusion of bentonite to improve the performance of geopolymer concrete with NCA and RCA are rather scarce. The present study, therefore, attempts to present the effect of bentonite on various properties of GNAC and GRAC mixes, namely, workability, air content, fresh density, compressive strength, split tensile strength, water absorption, chloride migration coefficient, and sulfuric acid attack resistance. The high-calcium fly ash (HCFA) was used as the primary source material to produce the geopolymer concrete; therefore, it can set and harden at ambient temperature. Bentonite was used as a partial replacement (0%, 10%, 15%, and 20%) for fly ash in both the GNAC and GRAC. A comparison between the behavior of both GNAC and GRAC mixes with different levels of bentonite is also presented to highlight their significant features.

### Research Significance

Despite several environmental benefits of GRAC, it has inferior engineering properties due to weak internal structure as compared to geopolymer natural aggregate concrete (GNAC). Low-calcium bentonite has been extensively used in the literature to improve the properties of conventional cementitious composites. However, the studies highlighting the effect of bentonite on the mechanical and durability properties of geopolymer concrete with RCA are rather scarce. In this study, an effort has been made to present the effect of bentonite on various properties of both GNAC and GRAC mixes, namely, workability, air content, fresh density, compressive strength, split tensile strength, water absorption, chloride migration coefficient, and sulfuric acid attack resistance.

## 2. Experimental Program

In the experimental program, a total of 10 mixes were designed to study the influence of bentonite clay content on the rheological, mechanical, and durability properties of geopolymer concrete containing natural and recycled coarse aggregates. The HCFA was used as the primary source material to produce the geopolymer binder. Low-calcium bentonite clay was obtained from its natural deposits in Jahangira (Nowshera), Pakistan, for partial replacement of HCFA in geopolymer concrete. The mixes were divided into two groups, GNAC and GRAC, based on the aggregates used, i.e., NCA and RCA, respectively. For studying the influence of bentonite clay, HCFA was replaced by bentonite clay by 0%, 5%, 10%, 15%, and 20% (by weight) in both the GNAC and GRAC mixes. A series of experiments were conducted to study the effect of bentonite content on rheological, mechanical, and durability properties of both GNAC and GRAC mixes. The fresh properties of the concrete mixes such as workability, fresh density, and air content were measured soon after completion of the mixing process. The mechanical properties of the mixes were evaluated by compressive and split tensile strength tests. The durability of the GNAC and GRAC mixes was evaluated by water absorption, chloride migration coefficient, and sulfuric acid resistance tests of specimens.

### 2.1. Materials and Methods

#### 2.1.1. Fly Ash and Bentonite

In this study, HCFA was used as the primary source material to produce geopolymer concrete. The chemical composition of HCFA is given in Table 1. For partial replacement of HCFA, low-calcium bentonite clay was obtained from its natural deposits in Jahangira (Nowshera), Pakistan. The feasibility of this bentonite as a partial replacement of OPC (up to 25% by weight) in conventional concrete has been verified by the previous studies [41,42]. 

**Figure 1:**
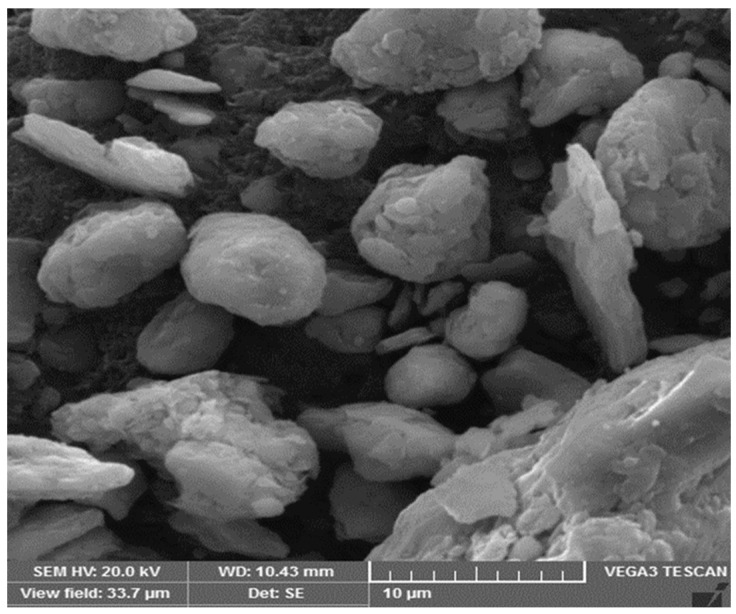
SEM image of low-calcium bentonite.

The raw bentonite obtained from the natural deposits was processed by wet grinding to make it pass through a 325 µm sieve. The XRF analysis confirmed that the bentonite used is low-calcium pozzolana as per ASTM C618-17a [51]. Moreover, scanning electron microscopic (SEM) (VEGA3 TE model, TESCAN, Brno, Czech Republic) imaging of bentonite revealed spherical and thick flake-shaped particles, as shown in Figure 1. The chemical composition and physical properties of bentonite are shown in Table 1. It can be seen from the table that the specific surface area of bentonite used in this study is higher than the OPC. This is due to the wet grinding process performed on the bentonite, as mentioned above. The comparison of the surface area of bentonite and OPC (Table 1) depicts more fineness of the bentonite than the OPC. The particle size of bentonite varies between 0.2 and 200 μm while the particle size range for cement is 0.2 to 100 μm. Fly ash consists of silt-sized particles which are generally spherical, typically ranging in size between 10 and 100 microns.

#### 2.1.2. Alkaline Activation Solution

The solutions of sodium silicate (SS) and sodium hydroxide (SH) were mixed in a ratio of 1:2 (SS/SH = 2.0) to make the alkaline activation solution. This solution was kept as 40% of the total binder quantity. The solution of SH of 12 M molarity was prepared by mixing 98–99% pure pellets of SH (with a specific gravity of 2.10) in water. It was prepared 24 h before the application due to the heat development following the exothermic reaction. The SS solution with a ratio of SiO_2_/Na_2_O = 2.0 was obtained from a local company (Sika, Rawalpindi, Pakistan). It was composed of Na_2_O, SiO_2_, and H_2_O in 15.06%, 29.95%, and 54.99% by mass, respectively. The properties of the SS solution are shown in Table 2.

#### 2.1.3. Aggregates

**Figure 2:**
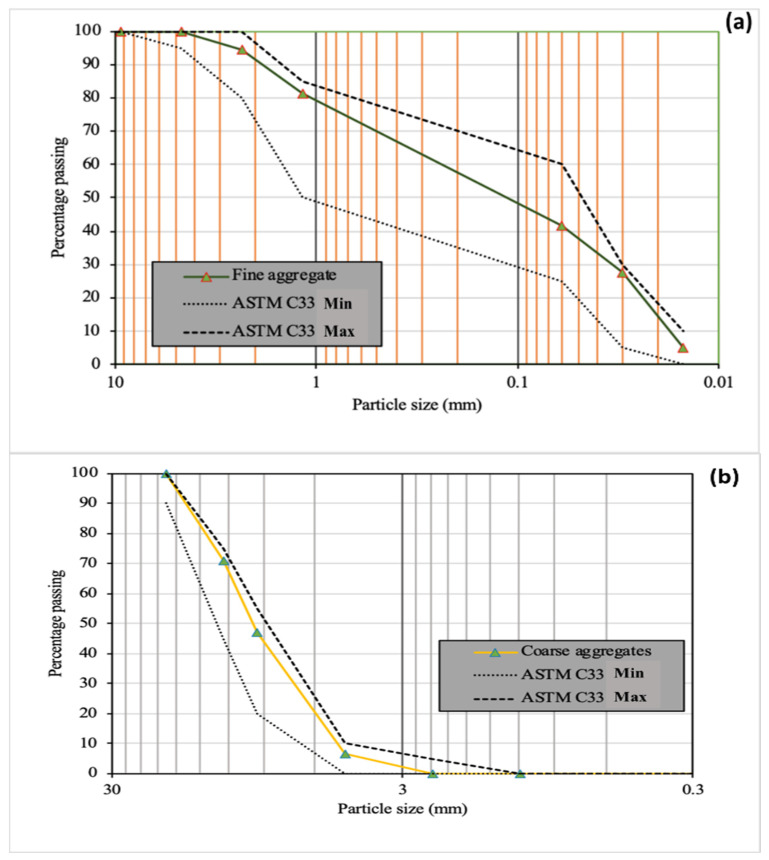
The gradation curves of (**a**) fine aggregates (**b**) RCA and NCA.

The natural river sand from Lawrencepur (Attock) Pakistan was used as fine aggregates in surface dry condition (SSD) conforming to ASTM C128-15 [52] for both GNAC and GRAC. The gradation of the fine aggregates falls under the acceptable limits, as shown in Figure 2a. The NCA with a maximum size of 19 mm were obtained from the Margallah quarry source in Pakistan to produce GNAC. The RCA were prepared by crushing laboratory tested concrete specimens with a compressive strength of 30–35 MPa. The gradation curve of both NCA and RCA is kept the same, as shown in Figure 2b. The properties of fine and coarse aggregates are shown in Table 3.

### 2.2. Mix Proportions

In this study, the mixes were divided into two groups, GNAC and GRAC, based on the aggregates used, NCA and RCA, respectively, as shown in Table 4. The HCFA was used as the primary source material to produce the geopolymer binder. For studying the influence of bentonite clay, HCFA was replaced by bentonite clay by 0%, 5%, 10%, 15%, and 20% (by weight) in both the GNAC and GRAC. The GNAC and GRAC specimens with 0% replacement level of bentonite are considered as primary (PC) and secondary (SC) control mixes, respectively. The quantity of the binder (425 kg/m^3^) was kept the same for all the mixes, and calculations for the replacement of HCFA by the bentonite were performed as per their mass ratio in the mix. The proportions of each mix are shown in Table 5. All the mixes are proportioned for a target slump of 75 mm to maintain the workability. An additional 20 kg/m^3^ of water was used in the GRAC mixes to overcome the water absorption of RCA. 

To maintain the workability of the mixes due to the negative effect of the increasing level of bentonite on the slump, superplasticizer was used. The reduction in the slump value is due to a relative increase in the angular and flaky shapes (having more surface area) of the bentonite particles as compared to the spherical shaped particles (having more flow-ability) of FA. Due to the larger surface area of the bentonite particles, more water is required in wetting the bentonite particles.

### 2.3. Mixing and Curing Process

All the mixes were prepared in a 400 L drum mixer following the conventional mixing procedure. The SH and SS were mixed in predetermined ratios to make an alkaline activator solution. This activity was performed about one hour before the mixing operation due to heat evolution resulting from the exothermic reaction. The mixing procedure and the time of mixing were kept the same for all the mixes. The sand was added at the start, followed by the binder materials (fly ash and bentonite) and coarse aggregates. After 3 min of dry mixing, the alkaline solution was added to the premixed dry ingredients. Then, water and superplasticizer were added, if required, and the mixing operation was continued for another 3 min to ensure homogeneity. The freshly mixed geopolymer concrete was poured into the molds of specimens. The compaction of specimens was carried out by the needle-type vibrator during casting. The specimens were kept at ambient temperature for one hour to enable setting and hardening prior to the heat curing [45]. Before curing the samples at 60 °C for two days, they were covered in plastic sheets to prevent loss of moisture due to evaporation during the process of curing at high temperature [30,31,45]. The specimens were then demolded, wrapped with plastic film, and placed in the curing room under the ambient environment with a temperature of 25 ± 2°C and relative humidity of 70 ± 5% until the testing age.

### 2.4. Testing Procedures

**Figure 3:**
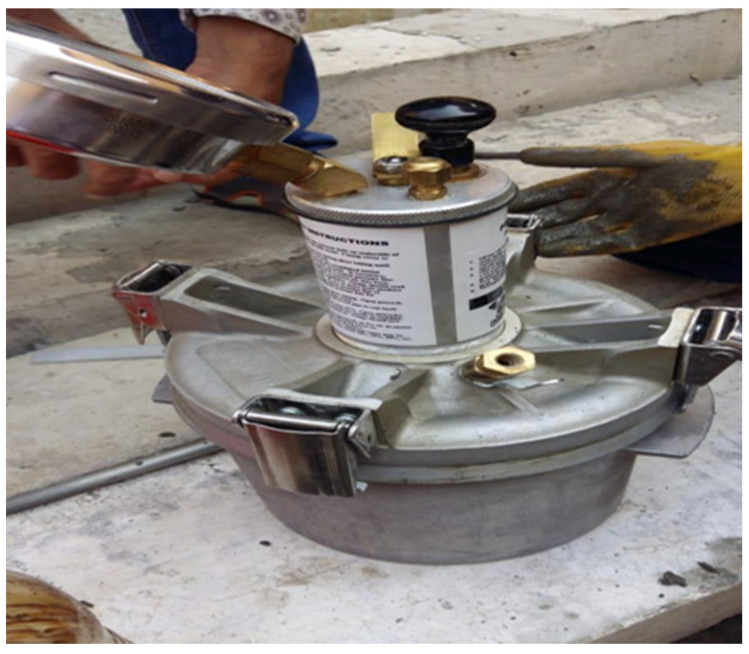
Air content apparatus.

The fresh properties of the concrete mixes such as workability, fresh density, and air content were measured soon after completion of the mixing process. The workability of the fresh mixes was measured by the slump cone test as per the ASTM C143/C143M-12 [53]. The fresh density of the GNAC and GRAC mixes was measured as per ASTM C138/C138M-17a wherein the mass of the concrete used to completely fill the container was divided by the volume of the cubical container. For this test, a cubical container of 150 mm was filled in three layers with fresh concrete. Each layer was compacted by 25 strokes of a steel rod to remove air voids [54]. The air content of freshly mixed geopolymer concrete mixes was evaluated by using the pressure method following the ASTM C231/C231M-10 [55] using the Forney apparatus shown in Figure 3. As per the mentioned standard, the base of the air content test apparatus was filled in three equal layers with a proper compaction via 25 strokes of a steel rod for each layer. After rodding, the apparatus was struck from outside to remove any remaining air voids. We fastened the top portion of the apparatus having air meter to its base containing concrete. The device was then pressurized via a hand pump until the air meter was stabilized. The stabilized reading was noted down and subtracted from aggregate correction factor to calculate the air content in the prepared mix. 

The mechanical properties of the mixes were evaluated by compressive and split tensile strength tests. The compressive strength test was performed on 150 × 150 mm cubes following BS EN 12390-3:2009 [56] at the age of 3, 28, and 90 days. The cubes tested for compression were placed on the face perpendicular to the cast face and the constantly increasing force at the rate of 0.6 MPa/s was applied until the cubes failed. The reading at which the cube failed was noted at the compressive strength of respective cube. The split tensile strength tests were performed on 150 × 300 mm^2^ cylinders according to ASTM C496/C496M-11 [57] at the age of 28 and 90 days. The cast cylinder was placed in in a jig for alignment and adjust bearing strips, and finally placed in compressive testing machine to apply load until the specimen failed. The loading rate was kept between 0.7 and 1.4 MPa/s and the reading gave us the load at which the specimen failed. This reading was incorporated into Equation (1) to derive the splitting tensile strength of the respective specimen.

(1) $$T=\frac{2P}{\pi LD}$$

where *T* is splitting tensile strength, *P* is load at which specimen failed, *L* is longitudinal length of specimen, and *D* is diameter of specimen 

**Figure 4:**
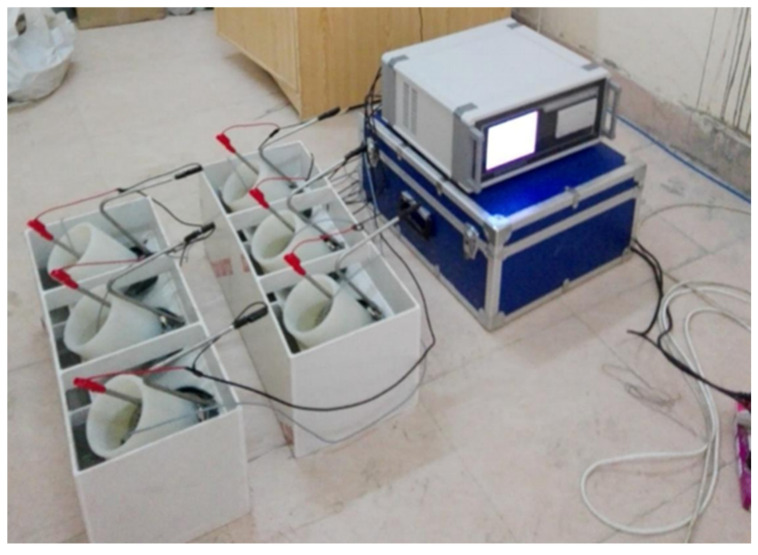
Experimental setup for chloride migration.

The durability of the GNAC and GRAC mixes was evaluated by measuring water absorption, chloride migration coefficient, and sulfuric acid resistance of specimens. The water absorption and chloride migration coefficient of the mixes were determined by 100 mm diameter discs of 50 mm thickness at the age of 90 days according to ASTM C948-81 [58] and NT Build 492 [59] (using SAMYON RCM apparatus with the test setup as shown in Figure 4), respectively. The water absorption was calculated by recording the weight of freshly cast specimen and weight of specimen after 90 days. Finally, the percentage increase in the weight of specimen was found and reported as percentage water absorbed by the respective specimen. The chloride migration test was performed on 90 days cured cylindrical specimens. The specimens are the pre-conditioned by placing them in vacuum chamber and then sealed to allow the penetration of chloride at the specimen ends. The sealed specimens were placed in cells with reservoirs having sodium hydroxide and sodium chloride solution at each end. To initiate chloride migration, a specific voltage was applied across each specimen. Finally, the specimens were taken out of cells and split using compression machine and sprayed split face with silver nitrate, which reacts with chloride to form a distinctive boundary identifying chloride penetration through the respective specimen. The depth of this distinctive boundary gave chloride migration. The cubical specimens of 100 mm were used to measure the sulfuric acid attack resistance. After demolding, the cubical specimens were placed in potable water for days [37]. These specimens were then submerged in 4% H2SO4 solution [36]. The specimens were taken out of the solution after 28, 56, and 90 days of immersion, and loss in mass of the sample was determined. It should be noted that the solution was changed after 14 days to keep the concentration of the acid stable. The number of specimens prepared to evaluate the different rheological, mechanical, and durability properties of GNAC and GRAC mixes are listed in Table 6.

## 3. Results and Discussions

### 3.1. Fresh Properties

#### 3.1.1. Workability

**Figure 5:**
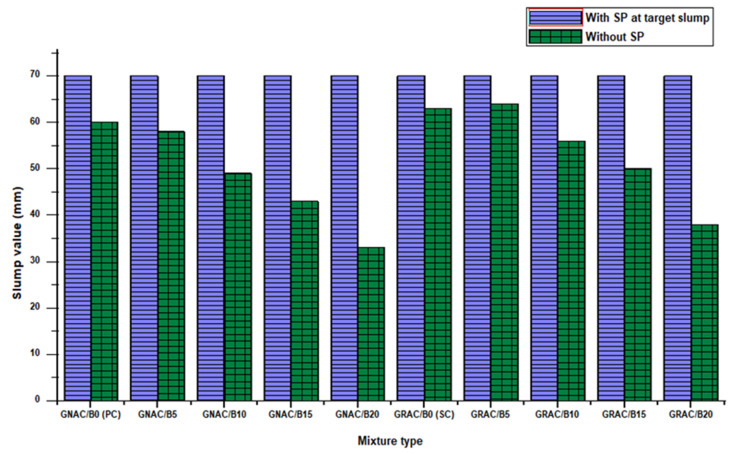
The slump values of GNAC and GRAC mixes.

The slump test was used to determine the workability of mixes. The test results of both GNAC and GRAC mixes are shown in Figure 5. It can be observed that the incorporation of bentonite has a negative effect on the workability of both GNAC and GRAC mixes. This reduction in a slump is due to the high specific surface area of the bentonite clay ash as compared to the fly ash particles [38]. The larger specific surface area of bentonite clay particles increases the water/solution demand in the mix to wet the particle surfaces completely, reducing the workability of the mix. Moreover, the particle size of bentonite is angular (see Figure 1) rather than circular in other supplementary cementitious materials (SCMs) (e.g., cenosphere) to provide a ball-bearing effect to increase workability [60]. 

Although the workability of the mix decreases by increasing the bentonite content, the mix remains cohesive. Superplasticizer (SP) was used to improve the lower slump values and to achieve the target slump. The target slump was selected as 70 mm for all the mixes. As depicted in Table 5, the SP dosage increases with a higher percentage replacement value of bentonite. The decreasing trend of slump values against increasing bentonite content is consistent with the previous studies [38,61]. It is important to notice that the reduction in workability is more pronounced in GNAC as compared to GRAC due to the presence of more free water in GRAC. A similar trend was also observed by Bilal et al. [41] and Kou et al. [6] in conventional concrete.

#### 3.1.2. Air Content

Air content is a property of fresh concrete that can play an important role in increasing or decreasing the concrete strength. The presence of air content in the concrete also determines its resistance to freeze and thaw cycles and other weathering agents. Generally, the strength of concrete decreases with an increase in the amount of air content.

**Figure 6:**
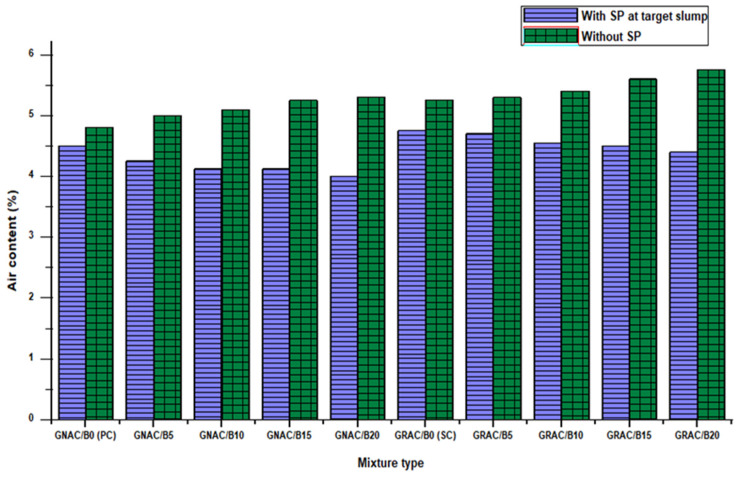
The amount of percent air content in the GNAC and GRAC mixes with and without SP.

The result of air content tests of the mixes is shown in Figure 6. It can be observed from Figure 6 that the GNAC mixes have lower air content than the corresponding GRAC mixes. The SC mix of GRAC has 5.5% more air content than the PC mix of GNAC. The amount of water used in the GRAC mixes to meet the water absorption demand of RCA is higher than their counterparts in the GNAC mixes, resulting in slightly higher workability than the GNAC mixes. However, the air contents of GRAC mixes are greater than the corresponding mixes of GNAC. This phenomenon can be attributed to the presence of unsaturated fractured zones in RCA, which are not connected to the permeable pores of aggregates [36].

It can be noticed from Figure 6 that the air content of the GNAC and GRAC mixes increased with an increase in the percentage replacement of bentonite when SP is not used to attain the target slump. This behavior can be due to the reduction in workability in the presence of bentonite. The less workable concrete easily allows the air to penetrate the fresh concrete, creating air voids.

On the other hand, when SP is used to achieve the target slump of mixes, reduction in the air content was observed with an increase in the bentonite replacement level for both the GRAC and GNAC mixes. The reduction in the air content can be ascribed to the fineness of bentonite particles that fill the spaces of air voids, resulting in a decrease in the volume of air voids in the mix [41]. A slight increase in the air content was also noted for the mixes containing RCA than the mixes containing natural aggregates, due to the porous structure of the RCA that can easily entrap air [41]. Due to the porous structure of RCA, the maximum increase in air content was found to be 6.4% in the GRAC mix with 20% bentonite content when compared to the PC without the SP addition. The GRAC mix with 20% bentonite content exhibits 4% higher air content than the PC mix at the target slump as can be observed from Figure 6. It can be inferred that the incorporation of bentonite in the GRAC mixes somehow overcome the deficits due to the RCA.

#### 3.1.3. Fresh Density

**Figure 7:**
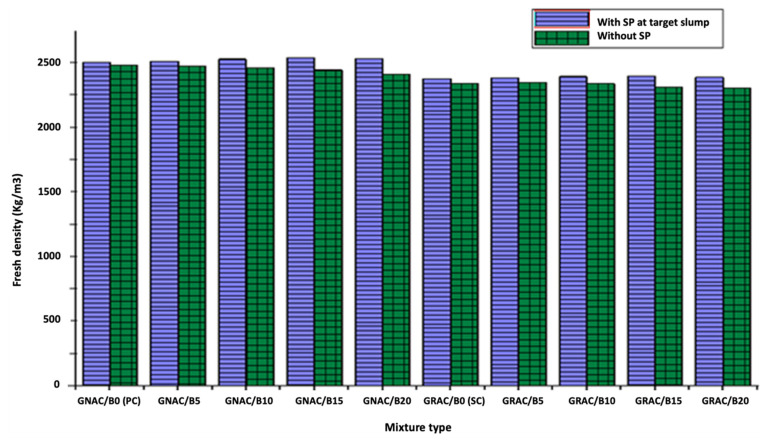
The fresh density of the GNAC and GRAC mixes with and without SP.

The fresh density of the GRAC and GNAC mixes (with and without the addition of SP) is shown in Figure 7. The fresh density of mixes (without SP) reduces with an increased percentage of bentonite for both the GRAC and GNAC mixes. 

At the highest replacement of bentonite (20%), a reduction in fresh density by 3% and 2% was observed in the GNAC and GRAC mixes, respectively, compared to their corresponding control mixes. The workability is an important factor that determines the density of concrete with a given compaction effort. Consequently, less workable concrete will result in a lower density of concrete. Therefore, it can be stated that the decrease in the fresh density of concrete mix is due to the lower workability of the mix resulting from an increase in the bentonite content in the mix. On the other hand, when SP is used to attain the target slump of mixes, the fresh density of both the GRAC and GNAC mixes is improved with an increase in the bentonite content when compared to their corresponding control mixes. This increase in the fresh density can be ascribed to the fineness of low-calcium bentonite particles that fill the pores of RCA, resulting in a reduction in air content. Moreover, the particles of bentonite are finer than the HCFA and can occupy the pore spaces of RCA and enhance the distribution of particle sizes in the geopolymer matrix of the mix [41,42]. 

### 3.2. Mechanical Properties 

#### 3.2.1. Compressive Strength

The compressive strength is considered to be a vital property of concrete for ascertaining the load-carrying ability of structural elements. The compressive strength of the GRAC mix generally depends on replacement level of RCA, the addition of mineral/chemical admixture, w/c ratio, concrete age, properties of parent materials, etc. The GRAC mix generally gives less compressive strength than the GNAC due to the higher porosity of RCA. However, the compressive strength of the GRAC can be improved by incorporating supplementary materials such as metakaolin, bentonite, silica fume, etc.

**Figure 8:**
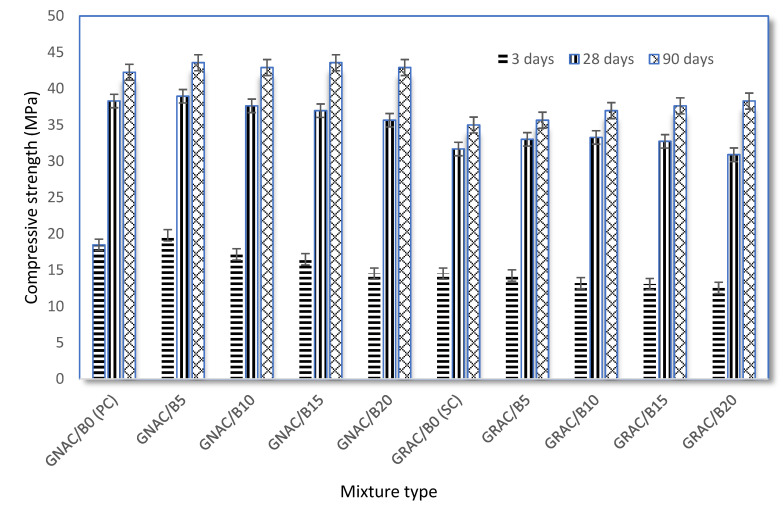
Compressive strength of the GNAC and GRAC mixes at 7, 28, and 90 days.

In the present study, the compressive strength of the GNAC and GRAC mixes with varying replacement levels of bentonite is evaluated at the age of 3, 28, and 90 days. The compressive strength tests were conducted on cubes according to BS EN 12390-3:2009 [56]. The strength of each mix was measured by conducting tests on three identical specimens of 150 × 150 × 150 mm size and the average strength of the three specimens is reported in Figure 8. It can be observed that an increase in the compressive strength was observed for 10% bentonite replacement than the corresponding control mixes. For example, 90-day compressive strength of GRAC/B10 and GNAC/B10 increased by 5.71% and 2.32%, respectively, as compared to the respective specimens containing 5% bentonite. When bentonite replacement is further increased from 10%, a decrease in the compressive strength was observed for both the GNAC and GRAC. For instance, 90-day compressive strength of GRAC/B15 and GNAC/B15 decreased by 8.11% and 4.52%, respectively, as compared to the specimens containing 10% bentonite. The probable explanation for this decreased compressive strength at the age of 3 and 28 days is slower pozzolanic reaction in the early ages, attributed to the formation of calcium silicate hydrate (C-S-H) and calcium aluminate silicate hydrate (CASH) gel at a slow speed. However, the rate of development of strength for bentonite mixes was found to be higher for older ages, i.e., 90 days for both the GNAC and GRAC mixes when compared to their corresponding control mixes. The control mixes SC and PC had lower strength than their corresponding group mixes. Similar findings were observed by Lima-Guerra et. al. [40] as well for conventional concrete. Moreover, it is interesting to note that the addition of bentonite has a more pronounced effect (increase or decrease) on the compressive strength of GRAC as compared to GNAC.

The RCA usually have old mortar attached to their surface, which makes them weaker and increases their water absorption capability. Due to the higher porosity of RCA, the GRAC requires additional water to compensate for RCA absorption. Although extra water helps the GRAC to maintain workability, it turns out to lower its hardened properties. Therefore, higher porosity of RCA can be one of the main reasons for the reduction in the compressive strength of the GRAC mixes [8,62]. Furthermore, due to high porosity and water absorption of RCA, the GRAC might have weak interfacial transition zone (ITZ) than the GNAC mixes [63].

**Figure 9:**
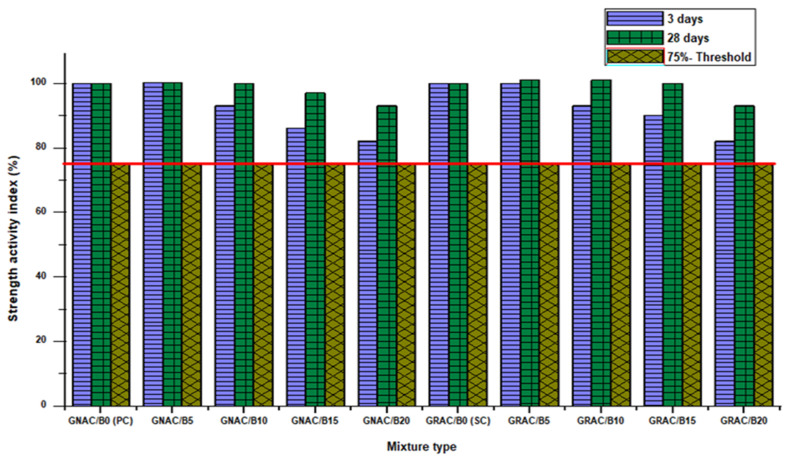
SAI of the GNAC and GRAC specimens.

The strength activity index (SAI) of each mix has been calculated according to ASTM C618-17a [51], as shown in Figure 9. SAI for each bentonite added mix, i.e., the GRAC and GNAC, should be higher than 75% (shown by red line as per ASTM C618-17a) of their relevant control mixes, i.e., SC and PC, respectively, at 28-day strength. It can be observed from Figure 9 that all the mixes of GNAC and GRAC have SAI well above the limit set by ASTM C618-17a. 

#### 3.2.2. Splitting Tensile Strength

Tensile strength is a mechanical property that is utilized in design aspects of concrete structures such as those associated with the initiation and propagation of cracks, shear, and anchorage of steel reinforcement in concrete [64]. The splitting tensile strength gives a specimen’s failure pattern under splitting action of loads rather than the true tensile strength of concrete. 

**Figure 10:**
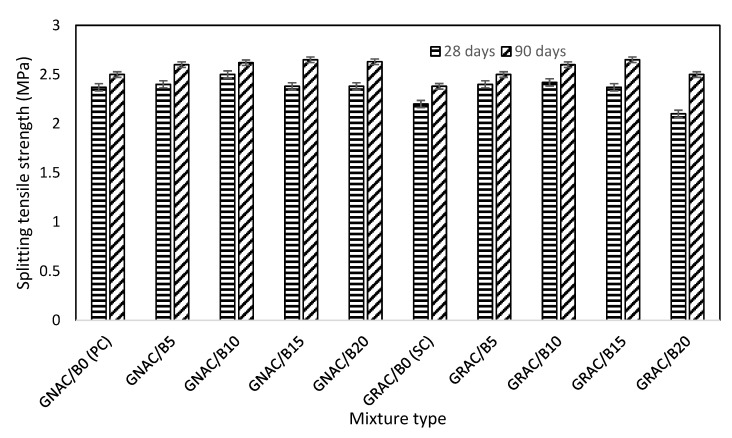
Splitting tensile strength of the GNAC and GRAC specimens at 28 and 90 days.

The splitting tensile strength tests were performed on 150 × 300 mm cylinders, after 28 and 90 days of casting according to ASTM C496/C496M-11 [57]. The tensile strength values of both the GNAC and GRAC mixes are shown in Figure 10. It can be seen that the effect of varying levels of bentonite on splitting tensile strength of the GRAC and GNAC mixes is similar to that of the compressive strength results. The difference in tensile strength values of both the mixes is evident, though not huge. The GRAC mixes have lower strength than their GNAC counterparts. As compared to the compressive strength, the tensile strength is relatively less affected by the replacement of natural coarse aggregates with recycled coarse aggregates.

The GRAC mixes contain porous and irregular-shaped aggregates that can be responsible for developing better bonding of RCA with the geopolymer matrix. This strong bonding is effective in resisting tensile stresses. The tensile strength of the GRAC and GNAC mixes was observed to be improving for bentonite replacement up to 10%, after which a decrease was observed. For instance, 90-day splitting tensile strength of GRAC/B10 and GNAC/B10 increased by 8.69% and 6.79%, respectively, as compared to the specimens containing 5% bentonite. The decreasing trend of tensile strength beyond 10% bentonite replacement is similar to the compressive strength results. For example, 90-day compressive strength of GRAC/B15 and GNAC/B15 decreased by 12.72% and 9.01%, respectively, as compared to the respective specimens containing 5% bentonite. However, all the bentonite-incorporated GRAC and GNAC mixes showed higher tensile strength than their corresponding control mixes at 90 days. Like compressive strength, bentonite has more pronounced effect (increase or decrease) on the splitting tensile strength of GRAC as compared to GNAC. Considering the performance criteria of mechanical properties, i.e., compressive strength and splitting tensile strength, 10 wt % of bentonite can be considered optimum content for both GNAC and GRAC mixes. The bentonite with 10 wt % can be incorporated to improve the mechanical properties of geopolymer concrete with RCA.

### 3.3. Durability Properties

#### 3.3.1. Water Absorption

Water absorption is a critical property in determining the durability of concrete since it measures water-accessible porosity of concrete. The water absorption of concrete controls the movement of outside moisture that could carry an aggressive chemical into the concrete.

**Figure 11:**
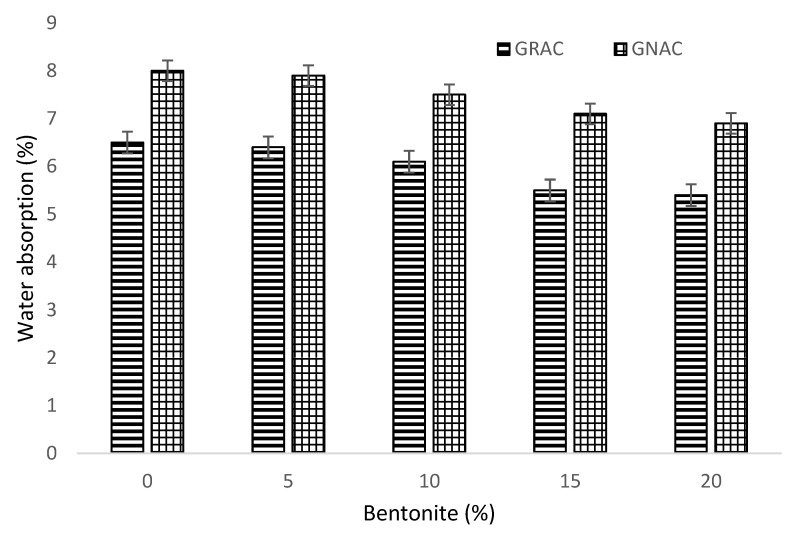
Percent water absorption of the GNAC and GRAC specimens with varying bentonite percentages.

There are various chemicals and salt solutions that can easily penetrate through pores of the concrete and can react with its ingredients to affect the material properties. The water absorption test was carried out on all mixes of GNAC and GRAC at the age of 90 days. The results obtained from the tests are shown in Figure 11. The incorporation of RCA in geopolymer concrete resulted in increased water absorption. The water absorption of GRAC mixes was observed to be increased by 29% than the GNAC mixes. This increase in water absorption can be attributed to the porous and permeable characteristics of old mortar attached to the surface of RCA that may have enhanced the connectivity between micro-channels in the microstructure of the material. Moreover, the old cement paste may have established routes for water to stream into the material [36,46].

The bentonite addition in both the GRAC and GNAC mixes has a positive effect on water absorption. The maximum reduction in the water absorption for both the GNAC and GRAC mixes was observed at 20% bentonite content. The water absorption of the GNAC mixes with 5%, 10%, 15%, and 20% bentonite decreased by 2.5%, 8%, 14%, and 18%, respectively, compared to the PC mix; the water absorption of the GRAC mixes with similar bentonite replacements decreased by 3.5%, 8%, 17%, and 20.5%, respectively, compared to the SC mix.

Due to the finer particle size of bentonite than the fly ash, it can have a uniform distribution in the mix matrix of both the GNAC and GRAC, thus providing a more compact and denser microstructure. In addition, the pozzolanic reactivity of bentonite may have resulted in the development of C-S-H gel in combination with geopolymeric gel that can provide a further denser microstructure of geopolymer matrix [65,66]. Therefore, it can be inferred that the incorporation of bentonite in both the GNAC and GRAC mixes is beneficial in terms of reducing the water absorption capabilities of the geopolymer matrix.

#### 3.3.2. Chloride Migration Coefficient 

The reinforced concrete’s durability and serviceability are greatly affected by chloride penetration from seawater or deicing salts. The reinforcing bars in the concrete corrode when chloride migration progresses on the steel bar surface. The formation of oxides at the steel surface results in increasing the volume of surrounding concrete, spalling and deteriorating the concrete cover. The determination of the chloride migration coefficient is an important technique to ascertain the ability of concrete to allow the penetration of chlorides.

**Figure 12:**
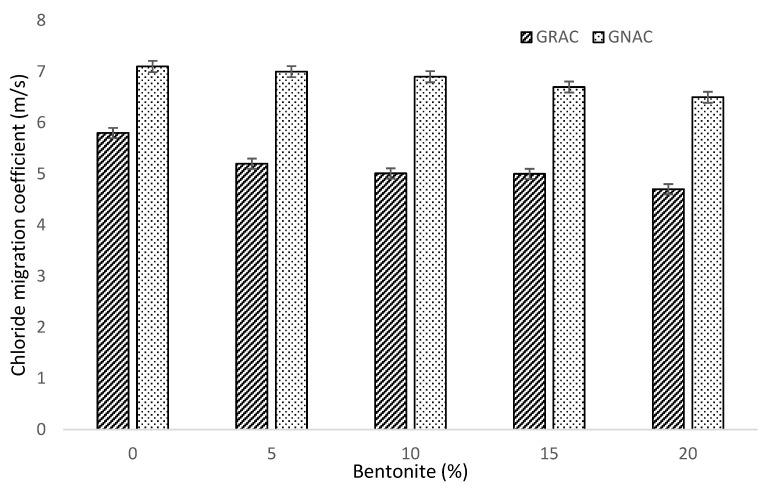
Chloride migration coefficient of the GNAC and GRAC specimens with varying bentonite percentages.

The results of the chloride migration test on all the mixes in terms of a coefficient are shown in Figure 12. The GRAC mixes have an appreciably higher coefficient of chloride migration due to their high porosity and poor interfacial transition zone (ITZ). The GRAC mixes showed 27% more penetration to chlorides than their counterparts of GNAC mixes. However, incorporation of bentonite in both the GRAC and GNAC mixes helped to improve the resistance against chloride migration. The better resistance with bentonite can be attributed to an improvement in impermeability and water tightness of concrete and enhancement of bonding capacity of bentonite with chlorides [41]. It is reported by Detwiler et al. [67] that supplementary materials, i.e., silica fume and GGBS, can be used to improve the resistance of concrete against chloride penetration. Nuaklong et al. [33] suggested the use of metakaolin as a supplementary material to improve the chloride penetration resistance of geopolymer concrete containing NA and RCA.

The inclusion of bentonite in the GNAC and GRAC mixes resulted in increasing the total alumina content, which in turn produced more calcium–alumina–silicate hydrates (C-A-S-H) in the concrete. The alumina content and C-A-S-H gel produce a protecting film in the concrete, which resisted the chloride penetration. While the chloride-carrying compounds enter into the pore structure of concrete, C-A-S-H reacts with these chlorides, producing Friedel’s salt by chemical reaction. Friedel’s salt increases the alkalinity of concrete by producing hydroxyl ions in the pore structure and acts as passivation for the steel reinforcement. 

#### 3.3.3. Sulfuric Acid Attack Resistance 

The nature of conventional concrete is alkaline, as indicated by its pH value. When the cement paste comes in contact with acids, its components break down. Therefore, in case of an acid attack on concrete, both hydrated and unhydrated cement compounds are dissolved along with the calcareous aggregates [68].

**Figure 13:**
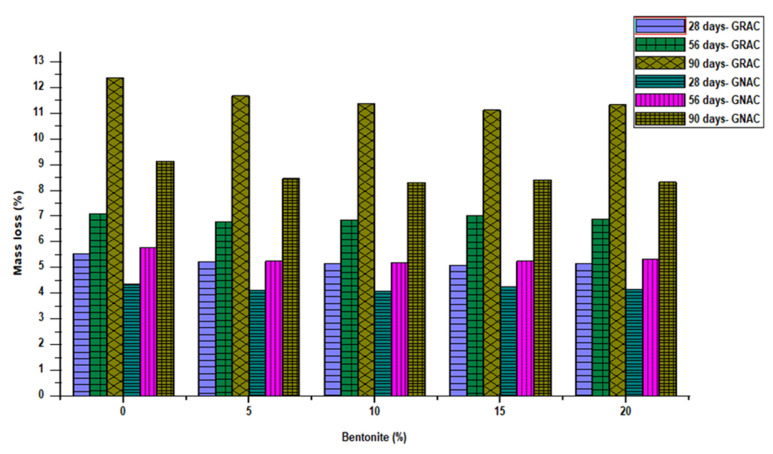
Percent mass loss due to sulfuric acid attack in the GNAC and GRAC specimens at varying bentonite percentages.

When the pH of the concrete is reduced, the ability of the binder to bind all the ingredients of concrete is affected and concrete will be more vulnerable to the acid attack. For an ideal concrete, the pH value should lie between 9 and 11. When the pH value is below 7, the acids may attack the concrete and make it unfit. The concrete structure starts deteriorating when water containing sulfuric acid penetrates the concrete. It produces sulfate ions which react with the portlandite to form gypsum. The gypsum produced reacts with calcium–aluminate hydrate to produce ettringite. The formation of the ettringite leads to the expansion of concrete, which creates internal pressure, resulting in deterioration. In this study, the resistance of concrete mixes against sulfuric acid is measured by determining mass loss in water containing 5% sulfuric acid and the results are shown in Figure 13. The specimens were submerged in 4% H_2_SO_4_ solution [41]. The specimens were taken out of the solution after 28, 56, and 90 days of immersion, and loss in mass of the cubes was noted. To keep the concentration of the sulfuric acid stable in the solution, the solution was changed every 14 days. It can be observed from the Figure 13 that loss in mass of cubical specimens increased with age. 

The GRAC mixes experienced a higher loss of mass in acid solution as compared to the GNAC, due to the mortar attached to the surface of the RCA. The inclusion of bentonite improved the resistance of both the GNAC and GRAC mixes. All the bentonite mixes exhibited a lower mass loss in the acid solution than their corresponding control mixes.

The higher resistance to acid solution of both the GNAC and GRAC mixes is due to the reduction in calcium oxide in concrete, owing to the replacement of bentonite. The control mixes PC and SC form more portlandite than the mixes with bentonite. The consumption of free portlandite by the bentonite particles in the pozzolanic reaction forms more CSH. This mechanism would have caused an improvement in acid attack resistance of both the GNAC and GRAC mixes. It can also be noticed that the inclusion of bentonite in the GRAC mixes resulted in an appreciable improvement in acid resistance. It can be noticed from Figure 13 that bentonite has a pronounced effect on GRAC mixes as compared to GNAC mixes because 15% bentonite incorporation reduces percentage mass loss (at 90 day) to 9% and 12% for GNAC and GRAC mixes, respectively. 

## 4. Conclusions

In this paper, we presented the results of an experimental program conducted to evaluate the influence of low-calcium bentonite content on the rheological, mechanical and durability properties of HCFA-based geopolymer concrete containing natural and recycled coarse aggregates. The mixes were divided into two groups, GNAC and GRAC, based on the aggregates used, i.e., NCA and RCA, respectively. For studying the influence of bentonite clay, HCFA was replaced by bentonite clay by 0%, 5%, 10%, 15%, and 20% (by weight) in both the GNAC and GRAC mixes. A series of experiments (workability, fresh density, air content, compressive strength, split tensile strength, water absorption, chloride migration coefficient, and acid resistance tests) were conducted to study the effect of bentonite content on rheological, mechanical, and durability properties of both GNAC and GRAC mixes. The following key conclusions have been drawn from this study:

1. The use of RCA in GRAC mixes results in increasing the workability as compared to corresponding GNAC mixes. The incorporation of bentonite has a detrimental effect on the workability of both the GRAC and GNAC mixes. The inclusion of bentonite reduced the workability of all concrete mixes; increased content of bentonite further intensified the reduction in workability. Therefore, to keep the same slump of all mixes, a small dosage of super plasticizer was added.
2. The air content of GNAC mixes was relatively lower as compared to the corresponding GRAC mixes. Air content of both GNAC and GRAC mixes increased with an increase in the percentage replacement of bentonite when SP is not used to attain the target slump. A significant reduction in the air content was observed with an increase in the bentonite content for both GRAC and GNAC mixes (with SP). The fresh density of GRAC mixes with RCA was lower when compared to the corresponding GNAC mixes with NCA. The fresh density of mixes (without SP) decreased with increased content of bentonite for both GRAC and GNAC mixes.
3. The use of RCA in GRAC mixes resulted in reduced mechanical properties compared to the counterparts of GNAC mixes. The replacement ratio of bentonite with HCFA had a significant effect on the strength properties of both GNAC and GRAC mixes. Replacement level of bentonite with up to 10% improved the strength properties of both GNAC and GRAC mixes. Beyond the 10% bentonite replacement level, the strength properties of both GNAC and GRAC mixes were effected.
4. The incorporation of RCA in GRAC mixes resulted in increasing the water absorption by 20–29% compared to the corresponding GNAC mixes. An appreciable reduction in the water absorption capacity of the GRAC mixes was observed at 90 days of specimen age for all replacement levels of bentonite. The maximum reduction in the water absorption for both the GNAC and GRAC mixes was observed at 20% bentonite content.
5. The GRAC mixes have appreciably higher coefficient of chloride migration than their counterparts of GNAC mixes. The mixes with bentonite addition showed greater resistance to chloride penetration than the control mixes (without bentonite). Furthermore, the GRAC mixes experienced higher loss of mass in acid solution as compared to the corresponding GNAC mixes due to the mortar attached to the surface of the RCA. The inclusion of bentonite resulted in improving the resistance of both the GNAC and GRAC mixes. All the bentonite mixes exhibited lower mass loss in the acid solution than their corresponding control mixes.

## Figures and Tables

**Table 1:** Chemical composition for Ordinary Portland cement, bentonite, and fly ash.

Oxides	OPC (% wt)	Bentonite (% wt)	Fly Ash (% wt)
SiO_2_	54.55	52.8	35.8
Al_2_O_3_	31.93	16.4	20.2
Fe_2_O_3_	3.12	5.8	11.4
MgO	1.42	1.4	1.80
CaO	4.64	4.6	14.3
Na_2_O	0.3	0.62	1.20
K2O	1.3	0.7	2.2
Relative density (g/cm^3^)	2.10	2.64	1.8
Specific surface area (cm^2^/gm)	3207	4900	3250
Loss on ignition (%)	1.30	9.6	0.57

**Table 2:** Properties of SS solution.

Entity	Specification
Color	Colorless
Density, kg/m^3^	1458
Total solid content	48 (% mass)

**Table 3:** Properties of fine and coarse aggregates.

Characteristic	Fine Aggregates	Coarse Aggregates	
		RCA	NCA
Maximum aggregate size (mm)	4.75	19.50	19.50
24-h water absorption (%)	0.59	6.25	0.63
10% Fine value (kN)	-	129.5	172.5
Dry rodded density (kg/m^3^)	1615	1377	1575

**Table 4:** Nomenclature for GNAC and GRAC mixes.

Type of Concrete	Specimen ID	Replacement of HCFA with Bentonite
Geopolymer natural aggregate concrete (GNAC)	GNAC/B0(PC)	0
	GNAC/B5	5%
	GNAC/B10	10%
	GNAC/B15	15%
	GNAC/B20	20%
Geopolymer recycled aggregate concrete (GRAC)	GRAC/B0(SC)	0
	GRAC/B5	5%
	GRAC/B10	10%
	GRAC/B15	15%
	GRAC/B20	20%

**Table 5:** Composition of GNAC and GRAC mixes.

Mix ID	Sand (kg/m^3^)	NCA (kg/m^3^)	RCA (kg/m^3^)	Fly Ash (kg/m^3^)	Bentonite (kg/m^3^)	NaOH (kg/m^3^)	Na_2_SiO_3_ (kg/m^3^)	Superplasticizer (SP) (kg/m^3^)
GNAC/B0 (PC)	640	1200		425	-	53	107	4
GNAC/B5	640	1200		404	21	53	107	4
GNAC/B10	640	1200		383	43	53	107	6
GNAC/B15	640	1200		361	64	53	107	8
GNAC/B20	640	1200		340	85	53	107	10
GRAC/B0 (SC)	640	-	1080	425	-	53	107	8
GRAC/B5	640	-	1080	404	21	53	107	10
GRAC/B10	640	-	1080	383	43	53	107	12
GRAC/B15	640	-	1080	361	64	53	107	14
GRAC/B20	640	-	1080	340	85	53	107	16

**Table 6:** Number of specimens conducted for each test.

Test	Testing Day				Total Specimens
	Day 3	Day 28	Day 56	Day 90	
	Number of Specimens				
Compressive strength	30	30	-	30	90
Splitting tensile strength	-	30	-	30	60
Water absorption	-	-	-	30	30
Chloride migration coefficient	-	-	-	30	30
Percentage mass loss	-	30	30	30	90

## Data Availability

All the data are available within this manuscript.

## Abbreviations

C&DW: Construction and demolition waste, C-A-S-H: calcium–alumina–silicate hydrate, CO_2_: Carbon dioxide, C-S-H: Calcium–silicate gel, FA: Fly ash, GGBS: Ground granulated blast furnace slag, GHG: Greenhouse gas, GNAC: Geopolymeric natural aggregate concrete, GPC: Geopolymer concrete, GRAC: Geopolymeric recycled aggregate concrete, HCFA: High-calcium fly ash, ITZ: Interfacial transition zone, NCA: Natural coarse aggregates, OPC: Ordinary Portland cement, PC: Primary control mix, RCA: Recycled coarse aggregates, RHA: Rice husk ash, SC: Secondary control, SAI: Strength activity index, SCMs: Supplementary cementitious materials, SEM: Scanning electron microscopic, SH: Sodium hydroxide, SP: Superplasticizer, SS: Sodium silicate, SSD: Surface dry condition.
